# Publisher Correction: Deacetylation of serine hydroxymethyl-transferase 2 by SIRT3 promotes colorectal carcinogenesis

**DOI:** 10.1038/s41467-019-08681-5

**Published:** 2019-02-12

**Authors:** Zhen Wei, Jinglue Song, Guanghui Wang, Ximao Cui, Jun Zheng, Yunlan Tang, Xinyuan Chen, Jixi Li, Long Cui, Chen-Ying Liu, Wei Yu

**Affiliations:** 1 State Key Laboratory of Genetic Engineering and Collaborative Innovation Center for Genetics and Development, School of Life Sciences and Zhongshan Hospital, Fudan University, Shanghai 200438, China; 2 Department of Colorectal and Anal Surgery, Xinhua Hospital, Shanghai Jiao Tong University School of Medicine, Shanghai 200092, China; 3 Shanghai Colorectal Cancer Research Center, Shanghai 200092, China

Correction to: *Nature Communications* 10.1038/s41467-018-06812-y. published online 26 Oct 2018.

The original version of this Article contained an error in the Data Availability Statement. The accession code indicated ‘53V’ and should have read ‘5X3V’. This has been corrected in both PDF and HTML versions of the Article.

