# Supplementary material for: Deacetylation of serine hydroxymethyl-transferase 2 by SIRT3 promotes colorectal carcinogenesis
Source: Nat Commun. 2018 Oct 26;9:4468. doi: 10.1038/s41467-018-06812-y (PMC6203763; doi:10.1038/s41467-018-06812-y)
Supplement: Supplementary file 1 — Supplementary Information [file 41467_2018_6812_MOESM1_ESM.pdf]

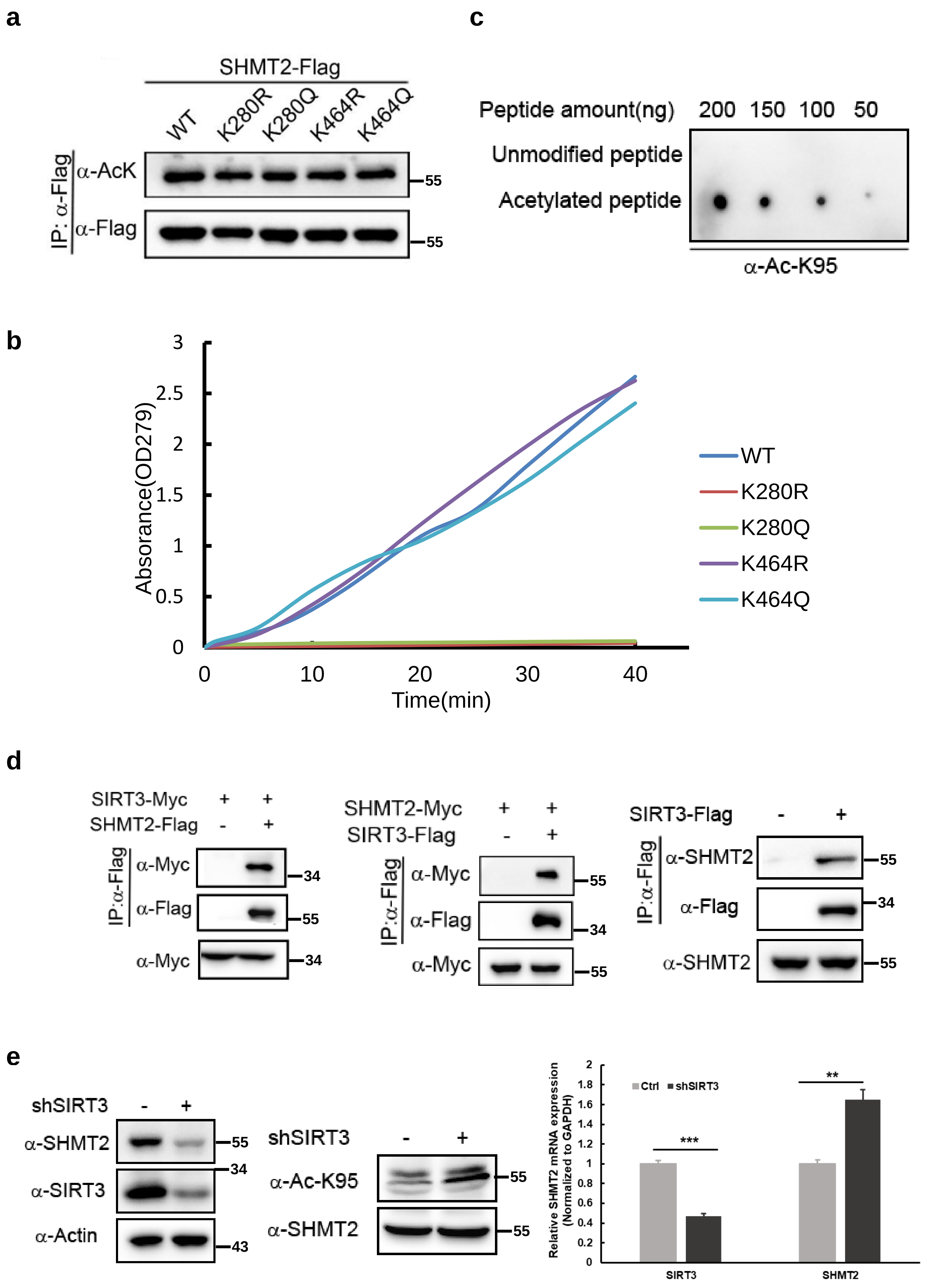

Supplementary Fig. 1 SHMT2 is acetylated at K95 and SIRT3 is the major deacetylase for SHMT2

Supplementary Fig. 1 SHMT2 is acetylated at K95 and SIRT3 is the major deacetylase for SHMT2

a Neither Lys 280 nor Lys 464 is the primary acetylation site of SHMT2. WT and K280R/Q, K464R/Q SHMT2-Flagged mutants were ectopically expressed into HeLa cells, and the proteins were immunoprecipitated before being subjected to western blot for acetylation analysis. **b** The SHMT2 K464R/Q mutant exhibited similar activity as the wild-type (WT) protein, and the K280R/Q mutant exhibited no activity because of its disturbed active site, as expected, which also showed that our method of assaying SHMT2 activity was feasible. All SHMT2 wildtype and mutants indicated were recombinantly expressed in E.coli and purified by nickel affinity chromatography and then performed activity assay with the same concentration. **c** Specificity of antibody against acetyl-K95 of SHMT2 as determined by dot blot assay. Nitrocellulose membrane was spotted with different amounts of unmodified peptide or acetyl-K95 peptide and probed with SHMT2-K95-Ac antibody. **d** SIRT3 interacts SHMT2. Interactions between SHMT2 and SIRT3 were determined when proteins were coexpressed with each other or endogenous SHMT2 was detected from immunoprecipitated exogenous SIRT3 in HeLa cells. **e** Depletion of SIRT3 increases SHMT2-K95-Ac and decreases SHMT2 protein levels but doesn’t decrease SHMT2 mRNA level in colorectal cancer cells. SIRT3 was stably knocked down in SW480 cells by shRNA. The knockdown efficiency, SHMT2 protein level and SHMT2-K95-Ac were determined by western blot. SIRT3 and SHMT2 mRNA levels were determined by Real-time PCR. Error bars represent $\pm$s.d. for triplicate experiments. The p value was calculated by student's t test. $^{**}P < 0.001$; $^{***}P < 0.001$.

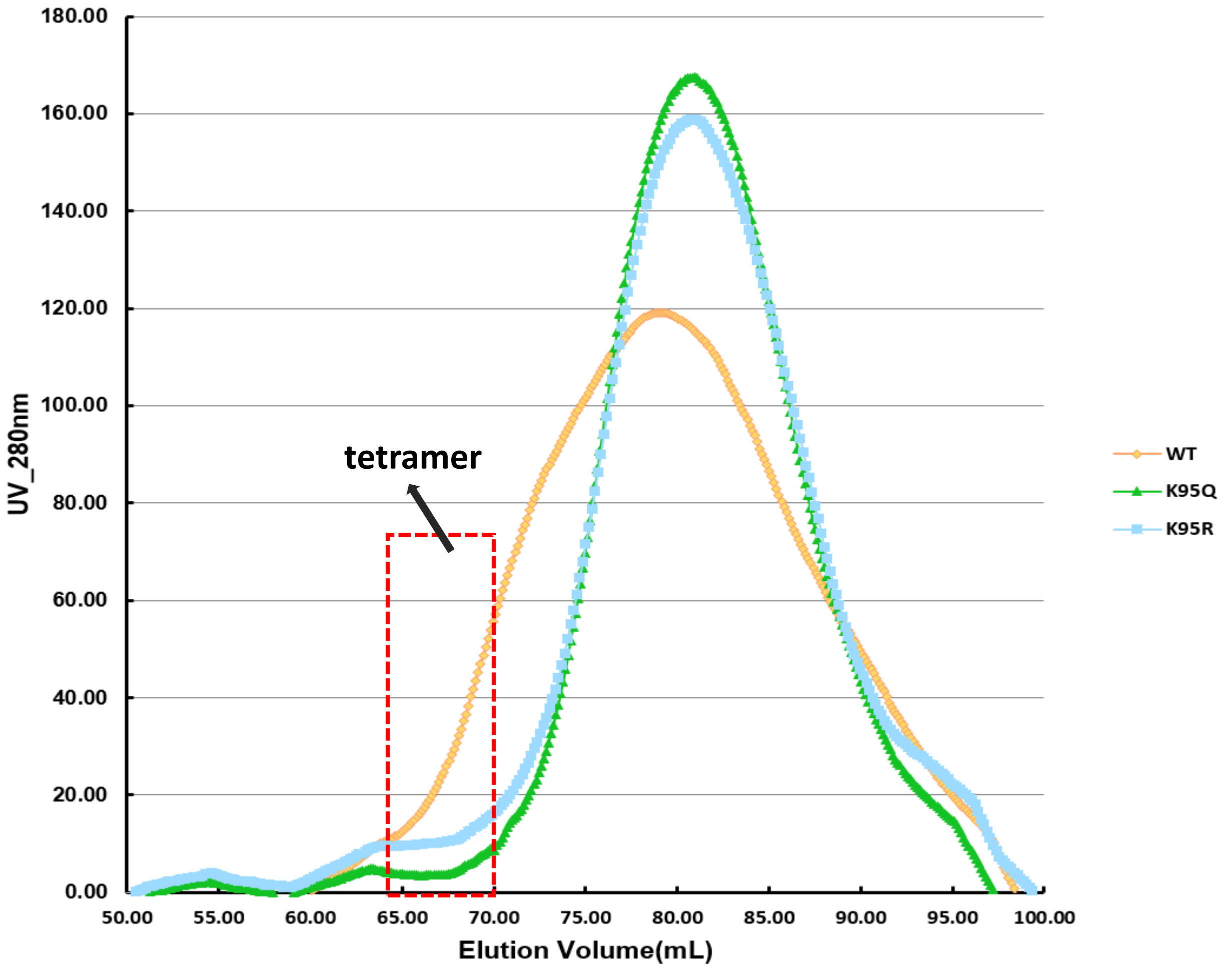

Supplementary Fig. 2 Size-exclusion chromatography

Absorbance profiles of SHMT2 WT, K95R/Q samples at 280 nm are shown. The arrows correspond to a range of molecular weight of the tetramer (around 250KD).

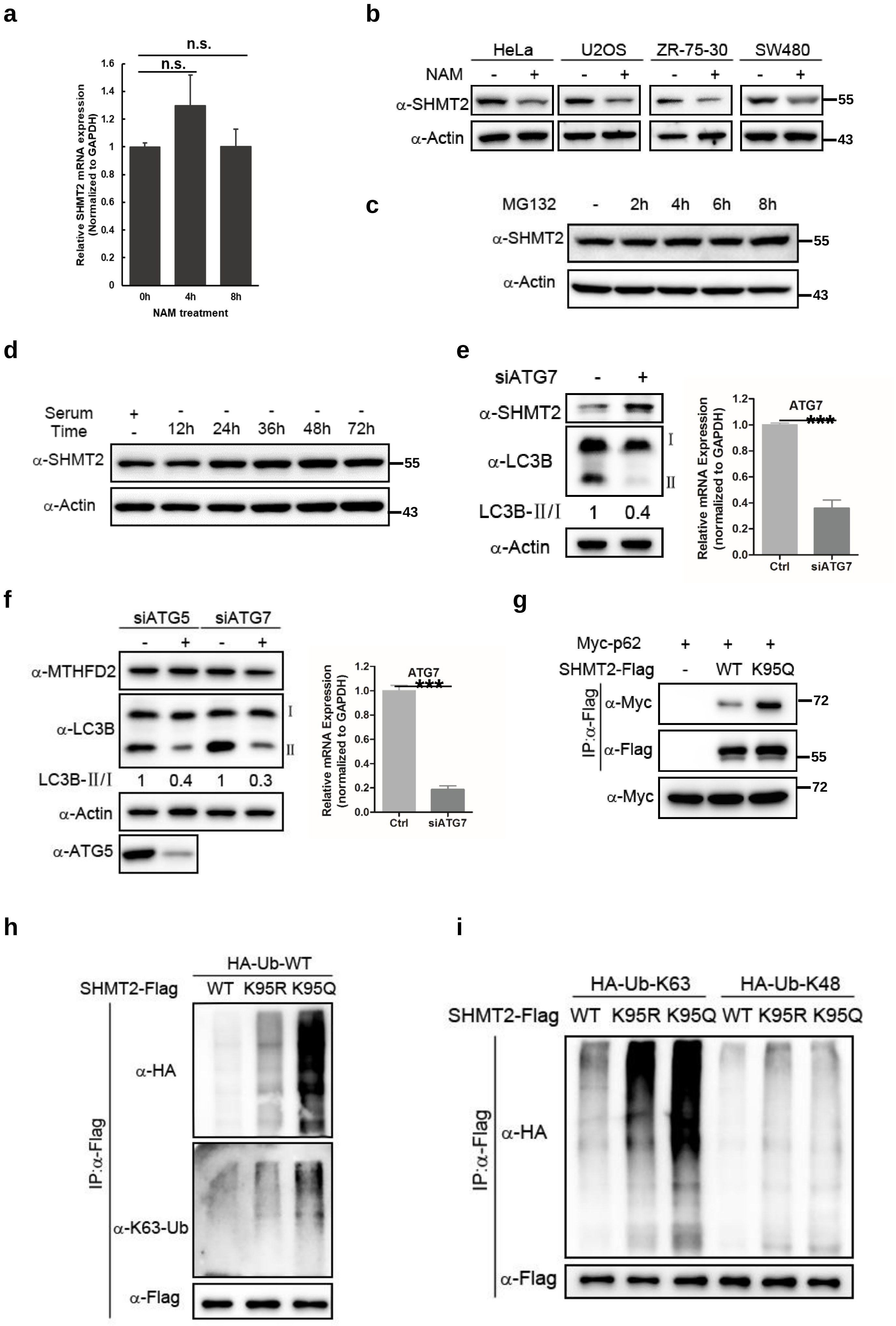

Supplementary Fig. 3 K95 acetylation promotes SHMT2 degradation through macroautophagy

Supplementary Fig. 3 K95 acetylation promotes SHMT2 degradation through macroautophagy

a Inhibition of deacetylases doesn't reduce SHMT2 mRNA level. SW620 cells were treated with or without NAM for 4h or 8h. The mRNA levels of SHMT2 were determined by Real-time PCR. Error bars represent ± s.d. for triplicate experiments. The p value was calculated by student's t test. n.s. not significant. **b** NAM treatment decreases SHMT2 protein level in different human cancer cells. The HeLa, U2OS, ZR-75-30 and SW480 cells lysate were prepared after NAM treatment, respectively. Endogenous SHMT2 protein levels were determined by western blot with indicated antibodies. **c** SHMT2 is not degraded by the ubiquitin-proteasome system (UPS). HeLa cells were treated with a proteasome inhibitor MG132(10μM) and the SHMT2 protein level was analyzed by western blot. **d** Serum withdrawal doesn't decrease SHMT2 protein level. SHMT2 level was determined by western blot after serum withdrawal for different lengths of time, as indicated in HeLa cells. SHMT2 may be degraded by the autophagic pathways not via CMA. **e** ATG7-knockdown increases SHMT2 protein level. HCT116 cells were transfected with siATG7 48h before harvest. The levels of SHMT2 and LC3B were analyzed by indicated antibodies. The decrease of LC3BⅡ/Ⅰindicates autophagic activity suppressed. The ATG7 knockdown efficiency was detected by real-time PCR. Mean values ±s.d. of relative mRNA expression of triplicate experiments are presented. ****P*<0.001. **f** ATG5 or ATG7 knockdown doesn't increase MTHFD2 protein level. **g** K95Q mutation increases SHMT2's interaction with macroautophagy receptor p62. Flag-tagged WT or K95Q SHMT2 with Myc-p62 were expressed in HCT116 cells, respectively and the proteins were immunoprecipitated by flag affinity agarose before being subjected to western blot for interaction analysis. **h** SHMT2 K95Q mutant binds more Ub chains. Flag-tagged SHMT2 WT, K95R and K95Q were co-transfected with HA-Ub WT in HEK293T cells. Cells were subjected to immunoprecipitated by flag affinity agarose and then western blot analysis with indicated antibodies. **i** SHMT2 K95Q mutant binds more K63-only-Ub chains not K48-only-Ub chains. Flag-tagged SHMT2 WT, K95R and K95Q were co-transfected with HA-Ub K63-only or K48-only in HEK293T cells. Cells were subjected to immunoprecipitated by flag affinity agarose and then western blot analysis with indicated antibodies.

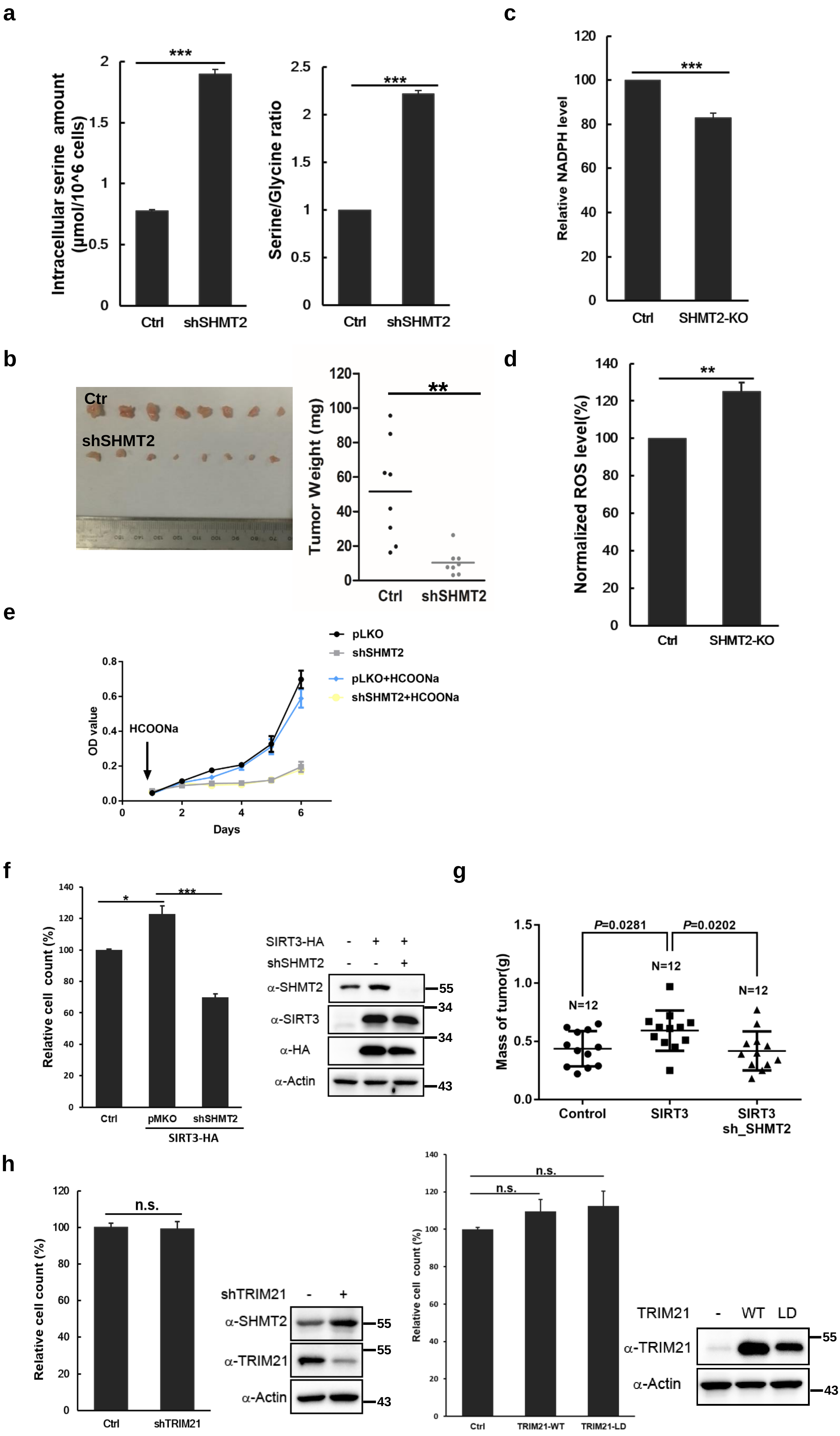

Supplementary Fig. 4 Loss of SHMT2 inhibits cell proliferation and tumor growth

Supplementary Fig. 4 Loss of SHMT2 inhibits cell proliferation and tumor growth

a Loss of SHMT2 increases intracellular serine level and serine/glycine ratio. SHMT2 knockdown and control SW480 cells were seeded in each well. Intracellular serine and glycine amount were measured by GCMS. Error bars represent ±s.d. for triplicate experiments. The p value was calculated by student's t test. **b** Loss of SHMT2 decreases tumor growth *in vivo*. Xenograft was performed using theSHMT2-knocking down and control SW480 cells as indicated. 21 days later, mice were sacrificed and tumor weight was measured. The p value was calculated by student's t test. **c** Loss of SHMT2 decreases cellular NADPH level. Intracellular NADPH was determined in SHMT2 knockout and control HCT116 cells by using NAD(P)H-Glo Detection System. Error bars represent ± s.d. for quadruple experiments. The p value was calculated by student's t test. **d** Loss of SHMT2 increases cellular ROS level. SHMT2 knockout and control HCT116 cells were seeded in confocal dishes and ROS level was measured by adding 10μM H2DCF-DA. Fluorescent strength per unit area was quantified using the ImageJ software, followed by statistical analysis. **e** Formate doesn't rescue the proliferation of SHMT2-knockdown SW480 cells. 1mM HCOONa was added into the culture medium. The proliferation of SHMT2-knockdown and control SW480 cells were detected by CCK8 assay. **f** Overexpressing SIRT3 increases cell proliferation through SHMT2. Stable SIRT3-overexpressing, SIRT3-overexpressing with SHMT2 knockdown and control HCT116 cells were seeded in 12-well plates and counted after five days. The overexpression and knockdown efficiency were determined by western blot. **g** Overexpressing SIRT3 increases tumor growth through SHMT2. Indicated cells in **f** were used for xenograft assay. **h** TRIM21 overexpression or knockdown doesn't affect CRC cells' proliferation. TRIM21 knockdown(left) and overexpression(WT or ligase-dead (LD) mutant) (right) stable SW480 cells were established and were seeded in 12-well plates and counted after five days. The overexpression and knockdown efficiency were determined by western blot. For all figures, $*P < 0.05$; $**P < 0.001$; $***P < 0.001$.

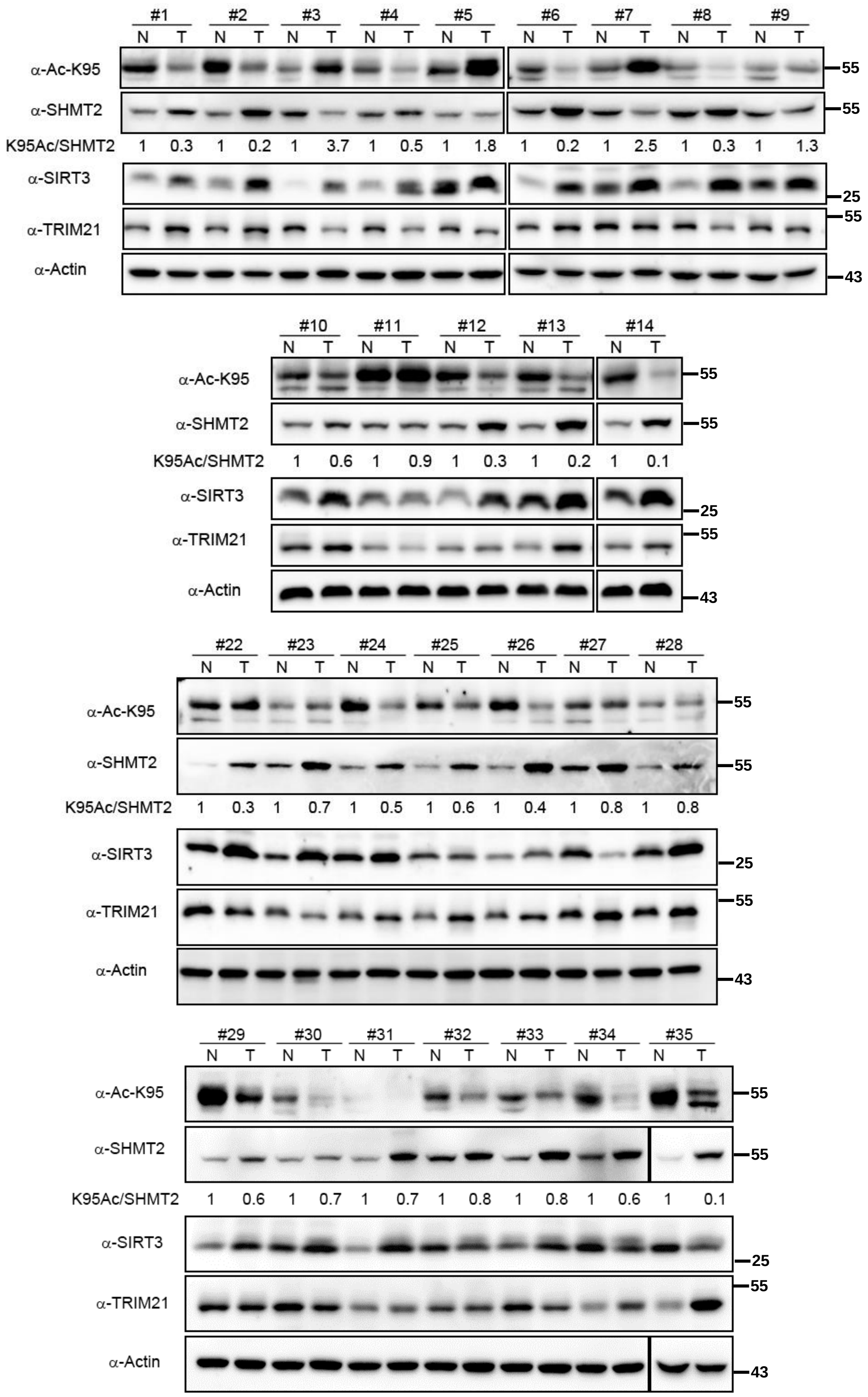

Supplementary Fig. 5 K95-acetylation of SHMT2 is downregulated in CRC

In total, 35 pairs of tumor tissues (T) and adjacent normal tissues (N) were lysed. Protein levels of SHMT2, SHMT2-K95-Ac, SIRT3 and TRIM21 were determined by direct western blot. Relative protein levels were normalized by β-actin. The other 28 pairs of samples(except for seven pairs shown in Fig 6a) were shown.

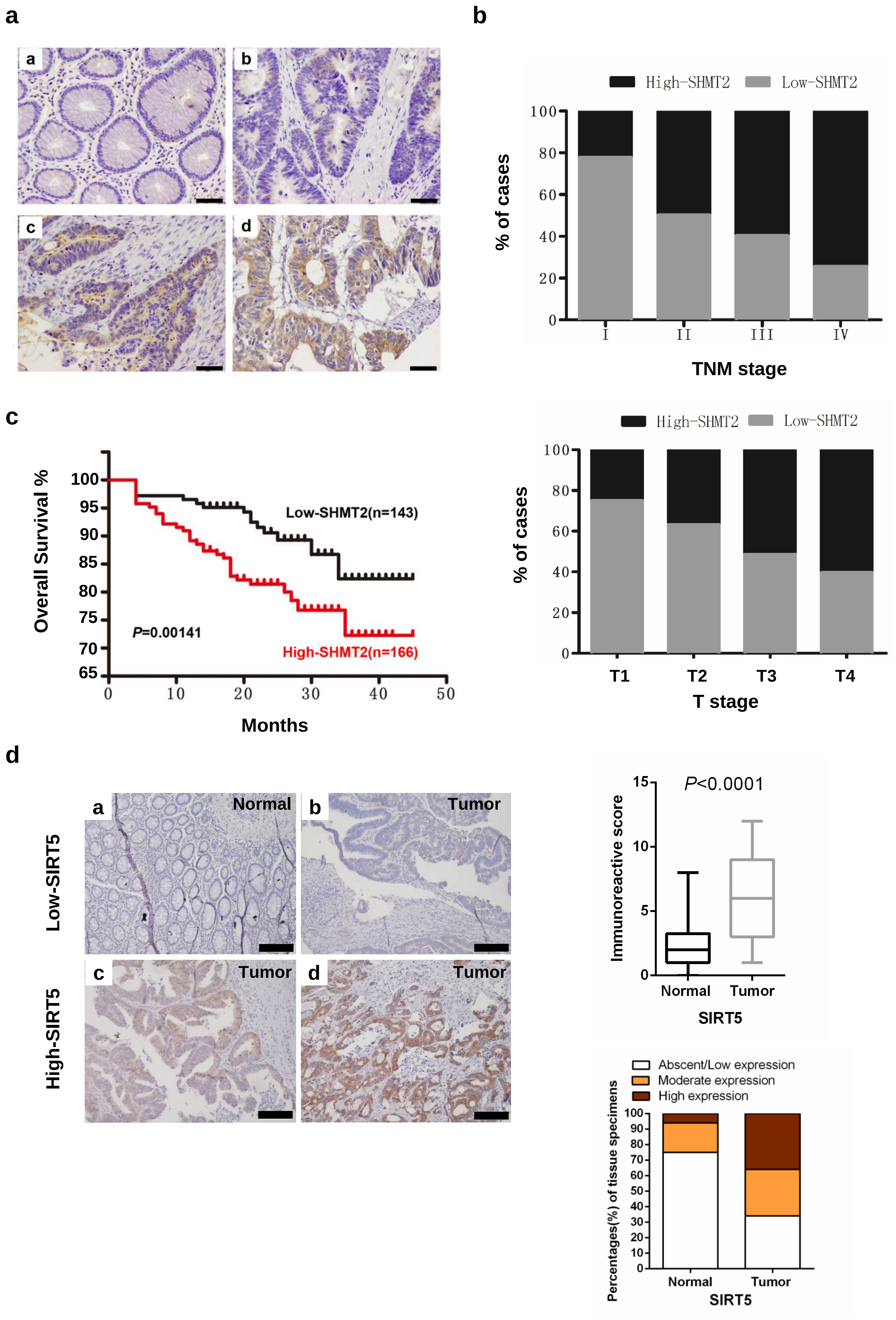

Supplementary Fig. 6 SHMT2 is overexpression in CRC

Supplementary Fig. 6 SHMT2 is overexpression in CRC

a Tissue-microarray analysis (TMA) of the SHMT2 protein expression level in 309 paired CRC and normal colon specimens. Scores indicate SHMT2 levels in representative tissues as determined by IHC staining. The scores were calculated by intensity and percentage of stained cells. (a. Normal colon specimen) (b, c, d. CRC specimens). Tumor expression level was diverging into low-(Score 0-4) and high-(Score 6-12) expression subgroups. Scale bars: 100 μm. **b** The patients with high SHMT2 expression have higher TNM stage and tumor stage. **c** The patients with high SHMT2 expression(n=166) have poorer overall survival compared with low SHMT2 expression (n=143). Significance was determined using Kaplan–Meier analyses. **d** Tissue-microarray analysis (TMA) of the SIRT5 protein expression level in 61 paired CRC and normal colon specimens. Scores indicate SIRT5 levels in representative tissues as determined by IHC staining. The scores were calculated by intensity and percentage of stained cells. (a. Normal colon specimen; b, c, d. CRC specimens: b. low-expression, c. medium-expression, d. high expression). Scale bars: 100 μm.

Supplementary Fig. 7 Uncropped scans of Western Blots

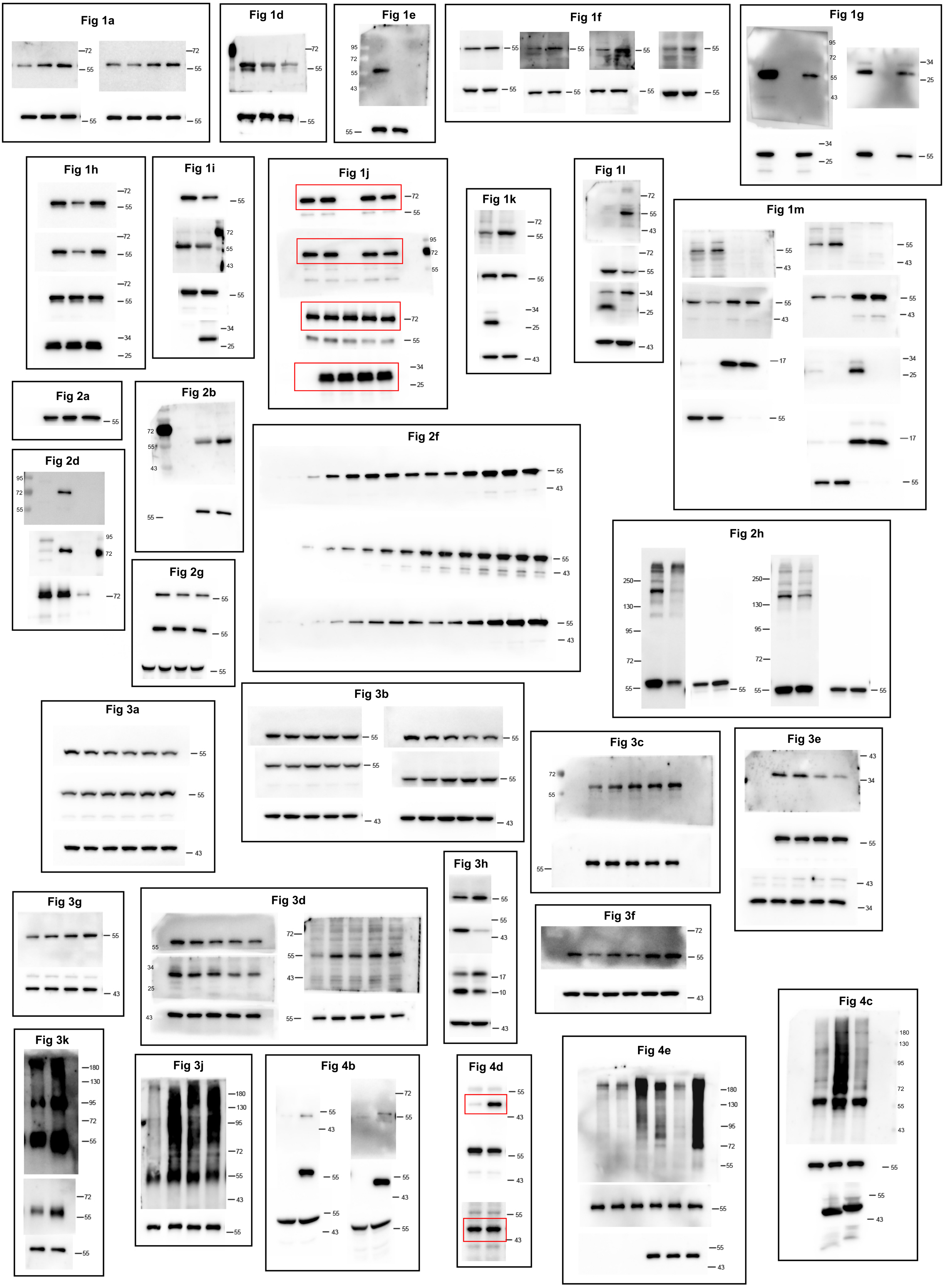

Supplementary Fig. 7 Uncropped scans of Western Blots (Continuation)

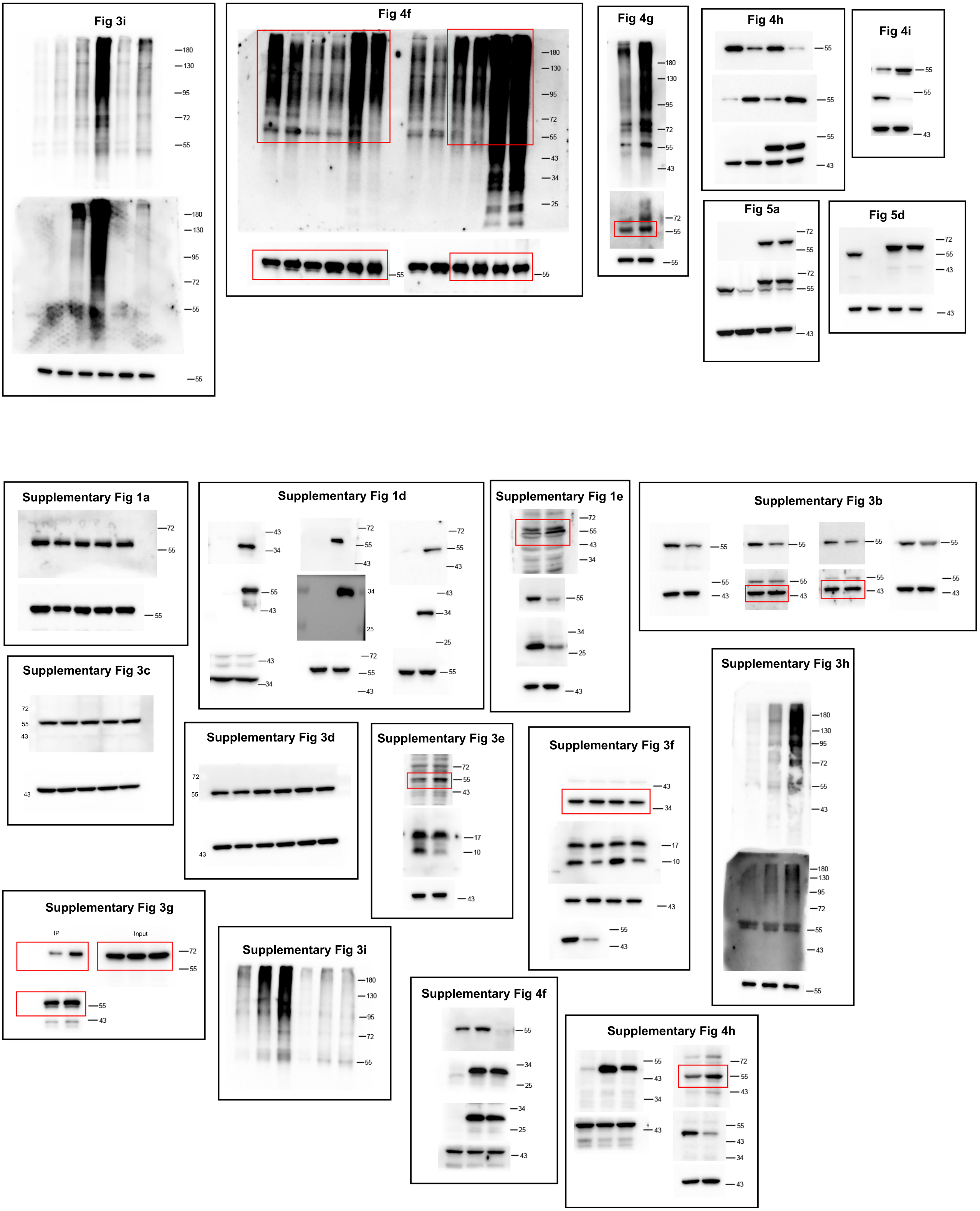

Supplementary Fig. 7 Uncropped scans of Western Blots (Continuation)

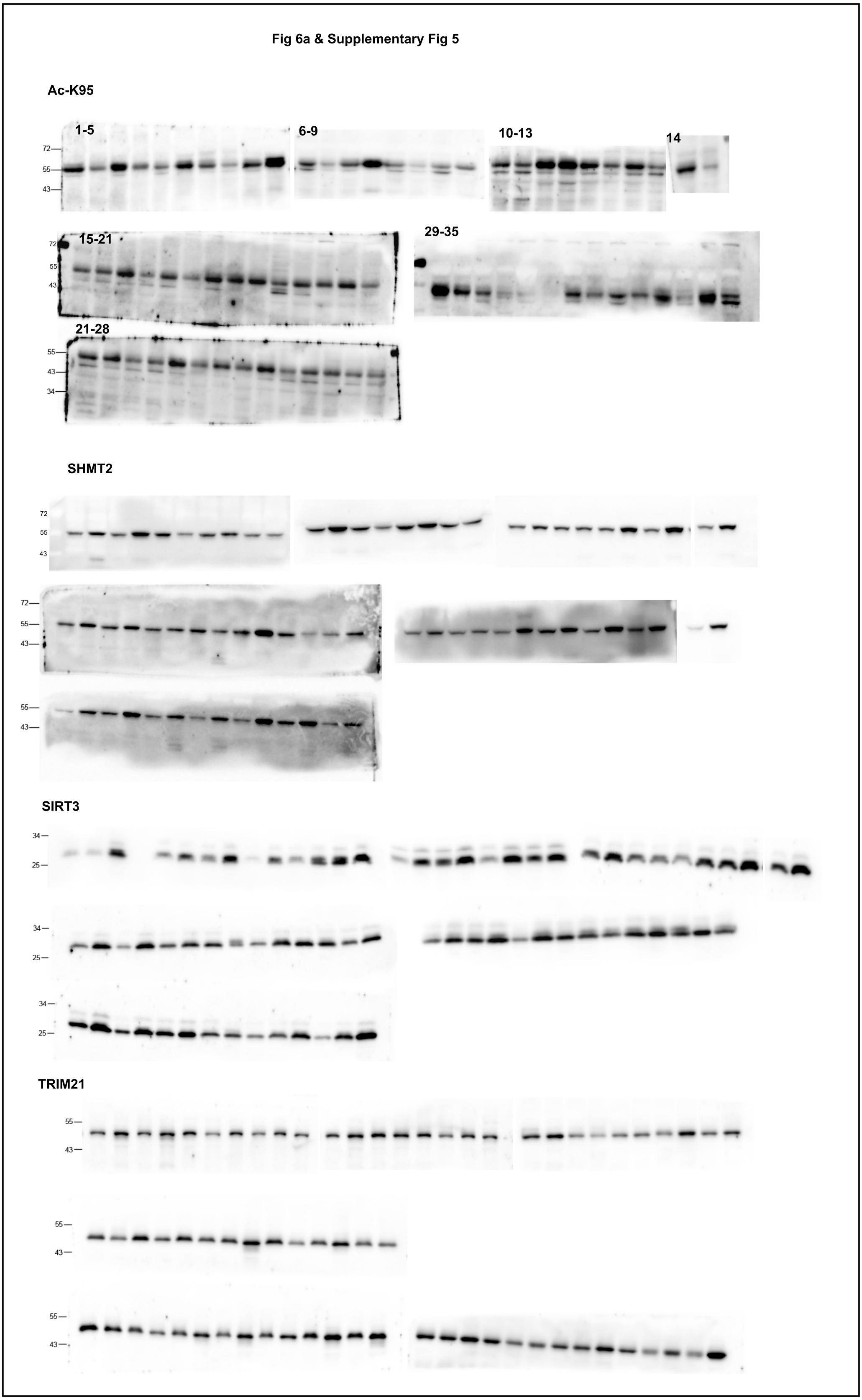

Table1. Statistics for data collection and refinement

**Data collection**	**SHMT2-K95R**
Wavelength (Å)	0.97776
Resolution (Å)	50.0-2.85 (2.952-2.85)[a]
Space group	P 6_5 2 2
Cell dimensions (Å, °)	a=158.680, b=158.680, c=209.376, α=β=90°, γ=120°
Unique reflections	27966
Completeness (%)	100 (100)
I/σ (I)	28.35 (2.6)
Redundancy	31.3 (21.5)
Wilson B-factor	45.37
CC1/2	0.837 (0.998)
**Refinement statistics**	
Reflections used in refinement	27966 (2746)
Reflections used for R-free	1414 (138)
R_{work} (%)[b]	17.95 (28.47)
R_{free} (%)[c]	23.26 (33.39)
No. residues	887
RMSD	
Bond lengths (Å)	0.011
Bond angles (Å)	1.65
Average B-factor ($Å^2$)	38.55
Ramachandran favored (%)	94.6
Ramachandran allowed (%)	5.4

A Values in parentheses are for the highest resolution shell. bRwork=∑||Fo|− |Fc||/∑|Fo|, where Fo and Fc are the observed and calculated structure factors for reflections, respectively. cRfree was calculated as Rwork using the 5% of reflections that were selected randomly and omitted from refinement.

Table2.Real-time PCR primers

Gene name	Sequence
TRIM21 (forward):	5'-TGAGTCCCCTGTAAAGCCAAA-3'
TRIM21 (reverse):	5'-TTGGCTAGCTGTCGATTGGG-3'
SIRT3 (forward):	5'-CAGTCTGCCAAAGACCCTTC -3'
SIRT3 (reverse):	5'-AACACAATGTCGGGCTTCAC-3'
SHMT2 (forward):	5'-AGTCTATGCCCTATAAGCTCAACCC-3'
SHMT2 (reverse):	5'-GCCGGAAAAGTCGAGCAGT-3'
ATG7 (forward):	5'-ACCTTGGGTTGCAATGTAGC-3'
ATG7 (reverse):	5'-CTCCTTGCTGCTTTGGTTTC-3'
GAPDH (forward):	5'-GCCACATCGCTCAGACACCA-3'
GAPDH (reverse):	5'-CTCAGCCTTGACGGTGCCAT-3'
